# Smartphones as Multimodal Communication Devices to Facilitate Clinical Knowledge Processes: Randomized Controlled Trial

**DOI:** 10.2196/jmir.2758

**Published:** 2013-11-27

**Authors:** Christoph Pimmer, Magdalena Mateescu, Carmen Zahn, Urs Genewein

**Affiliations:** ^1^Institute for Information SystemsSchool of BusinessUniversity of Applied Sciences and Arts Northwestern Switzerland FHNWBaselSwitzerland; ^2^Institute for Research and Development of Collaborative ProcessesSchool of Applied Psychology (APS)University of Applied Sciences and Arts Northwestern SwitzerlandOltenSwitzerland; ^3^Hightech Research CenterCranio-Maxillofacial SurgeryUniversity Hospital BaselBaselSwitzerland

**Keywords:** mobile health, mobile phone, telemedicine, educational technology, learning, problem solving, multimedia, audiovisual aids

## Abstract

**Background:**

Despite the widespread use and advancements of mobile technology that facilitate rich communication modes, there is little evidence demonstrating the value of smartphones for effective interclinician communication and knowledge processes.

**Objective:**

The objective of this study was to determine the effects of different synchronous smartphone-based modes of communication, such as (1) speech only, (2) speech and images, and (3) speech, images, and image annotation (guided noticing) on the recall and transfer of visually and verbally represented medical knowledge.

**Methods:**

The experiment was conducted from November 2011 to May 2012 at the University Hospital Basel (Switzerland) with 42 medical students in a master’s program. All participants analyzed a standardized case (a patient with a subcapital fracture of the fifth metacarpal bone) based on a radiological image, photographs of the hand, and textual descriptions, and were asked to consult a remote surgical specialist via a smartphone. Participants were randomly assigned to 3 experimental conditions/groups. In group 1, the specialist provided verbal explanations (speech only). In group 2, the specialist provided verbal explanations and displayed the radiological image and the photographs to the participants (speech and images). In group 3, the specialist provided verbal explanations, displayed the radiological image and the photographs, and annotated the radiological image by drawing structures/angle elements (speech, images, and image annotation). To assess knowledge recall, participants were asked to write brief summaries of the case (verbally represented knowledge) after the consultation and to re-analyze the diagnostic images (visually represented knowledge). To assess knowledge transfer, participants analyzed a similar case without specialist support.

**Results:**

Data analysis by ANOVA found that participants in groups 2 and 3 (images used) evaluated the support provided by the specialist as significantly more positive than group 1, the speech-only group (group 1: mean 4.08, SD 0.90; group 2: mean 4.73, SD 0.59; group 3: mean 4.93, SD 0.25; *F*_2,39_=6.76, *P*=.003; partial η^2^=0.26, 1–β=.90). However, significant positive effects on the recall and transfer of visually represented medical knowledge were only observed when the smartphone-based communication involved the combination of speech, images, and image annotation (group 3). There were no significant positive effects on the recall and transfer of visually represented knowledge between group 1 (speech only) and group 2 (speech and images). No significant differences were observed between the groups regarding verbally represented medical knowledge.

**Conclusions:**

The results show (1) the value of annotation functions for digital and mobile technology for interclinician communication and medical informatics, and (2) the use of guided noticing (the integration of speech, images, and image annotation) leads to significantly improved knowledge gains for visually represented knowledge. This is particularly valuable in situations involving complex visual subject matters, typical in clinical practice.

## Introduction

### Interclinician Communication and Mobile Phones

Interclinician communication is a key component of health care systems. The significance becomes clear in light of its impact on patient care: poor communication between clinicians results in enormous costs and, more importantly, a high number of adverse clinical outcomes and deaths [1-4]. Typical forms of communication between medical professionals are shaped by the particularities of clinical work and can be characterized as instant and synchronous [2,3,5], interdisciplinary and interprofessional (between actors holding different domains and levels of knowledge [6,7]), and mobile (ie, between physically/locally mobile hospital workers [3,8] who increasingly communicate using mobile technologies). In particular, cellphones and smartphones are becoming increasingly popular in clinical settings, with adoption rates of up to 98% [9-12]. According to recent reviews, in light of these characteristics and recent developments, there is little known about how mobile phone-based communication can contribute to effective interclinician communication [13-15]. In many of the existing studies that have explored mobile clinical communication technologies, the methodological design was reported to be of lower quality and based on users’ perceptions [13]. Additionally, only a few randomized controlled experiments were identified [13,16].

### Rich Communication Modes: Speech, Images, and Annotation

In recent publications, mobile phones have been considered as potentially efficient tools that enable instant location-independent communication [10,14,17-20]. In addition to supporting immediate communication in the form of speech, a number of studies have demonstrated how mobile phones and smartphones allow the ability to capture, exchange, and interpret images [14,21]. Comparisons to standard methods have demonstrated the suitability of mobile phones for use in assessment and diagnosis, such as when using computed tomography (CT), computed tomography angiography (CTA), and noncontrast computed tomography (NCCT) soft tissue or ophthalmic images [22-28]. A number of clinical information systems allow for the annotation of clinical images [29-31]; annotation refers to marking, drawing, highlighting, labeling, or otherwise describing (and enriching) aspects of visual material that should be the focus of attention. The creation of annotations is an essential part of clinical communication environments [32], and is considered a “fundamental task for clinicians, medical educators, or basic scientists” [30].

### Mobile Communication and Knowledge Exchange

The aim of synchronous interclinician communication involves the building of a shared understanding and a “just-in-time grounding” [2] between clinical actors with varying levels and domains of knowledge and expertise for the well-being of patients. During this process, knowledge is exchanged and new knowledge is created. Less knowledgeable actors learn from the communication and may transfer what they have learned to future patient cases. In other words, interclinician communication represents a valuable opportunity for medical actors to learn from one another for present and for future patient cases, similar to problem-based learning (eg, [33]). Regarding knowledge exchange and learning, some research emphasizes the value of using images in mobile phone-based communication. For example, it has been suggested that mobile image messaging can serve as an instructional tool that allows for instant feedback and further insights for less knowledgeable actors [34]. In a study involving doctor-to-doctor consultations that were based on images taken and sent by means of mobile phones (in the form of Multimedia Messaging Service, MMS), it was concluded that multimedia consultation had a positive effect on patient management and led to an improvement of the service. In addition, 86% of the residents reported “the multimedia information contributed to their ability to independently handle similar cases in [the] future” [35].

We conclude, despite the widespread use and advancements of mobile technology that enables rich communication modes, that there is a surprising paucity of evidence that demonstrates the actual value of mobile technology for effective interclinician communication, knowledge exchange, and learning.

### Objectives and Hypothesis

To address this research gap, we delineated 2 sets of hypotheses based on cognitive and sociocognitive science approaches. The first set of hypotheses relates to theories of dual coding and multimedia learning [36-39] and the second set relates to the notion of guided noticing in communication [40,41].

Concerning cognitive science, it has been shown that images support human information processing and understanding. This is not only because images convey different information than words (concrete vs abstract), but because text and images are processed in 2 different channels or modes [36-39]. The dual channel processing assumption asserts that humans have separate channels for processing words and images and that they use them both to construct coherent mental knowledge representations [36,38,39]. Empirical evidence has demonstrated picture superiority effects (ie, that people can recall information better from both words and images than from words alone) [37,42-44]. Moreover, a vast body of research on learning with multimedia [37,43] suggests that when words (written words or speech) and images are presented together and in a well-designed and integrated manner (eg, redundant and spatially contingent), they support the construction of coherent mental representations and, thus, learning (eg, learning about the functioning of physical systems) [45]. This idea is especially true for less knowledgeable learners (eg, [46]) and in situations in which test items were presented in image-based form [47]. However, research on dual coding and multimedia learning was primarily conducted in experimental studies by using simple content. There is little evidence that demonstrates learning effects in applied and more complex situations, such as that of interclinician communication and knowledge exchange. According to multimedia theory, we formulated our first assumption for the experimental design of mobile phone-based medical communication.

Mobile phone-based medical communication, in which speech and images are combined to enhance the understanding of complex subject matter, leads to a better understanding by a less knowledgeable actor when compared to the speech-only condition. Therefore, knowledge recall and transfer of verbally and visually represented knowledge should increase when compared to the speech-only condition. Therefore, in our specific case of mobile communication and less knowledgeable actors, we hypothesize that mobile communication involving integrated speech and images leads to better recall and better transfer of visually represented and verbally represented medical knowledge than the speech-only condition.

For our second set of hypotheses, we derived our assumptions from the concept of guided noticing. Guided noticing describes how specific digital technology affordances can be used in distributed collaborative settings to direct joint attention to notice specific elements in visual media (eg, videos, images). Guided noticing can involve highlighting (eg, annotation), naming, and commenting; thus, it allows the assignment of typological representations (culturally meaningful categories) to topological representations (eg, images). This is especially important when experts interpret visualizations in detail and explain their interpretations to others. Here, guided noticing can be a way to establish common ground [48] in communication between professionals (eg, in educational settings), for example, by using annotations. The creation of annotations is considered as a grounding or communication act by users (rather than designers) who intend to close gaps in common ground required to complete a task [32]. Guided noticing can also serve to develop domain expertise and a professional vision [49] (eg, the training of diagnostic skills with x-rays [41]). Professional vision was empirically investigated and characterized by Goodwin [49] as “...socially organized ways of seeing and understanding events that are answerable to the distinctive interests of a particular social group.” Case studies of professional practices (eg, developing coding schemes or highlighting) with domain experts (eg, archeologists in field research, lawyers in courtroom) were conducted analyzing these practices in great detail. Yet, empirical research on guided noticing [41] is still sparse. In our own previous research on history learning with advanced video tools, we identified episodes of guided noticing and analyzed them in relation to the successful acquisition of visual knowledge [50]. Our present study extends the existing qualitative research by adding original quantitative data on the effects of highlighting (by annotation) for guided noticing in the specific area of medical knowledge.

Accordingly, we formulated the assumption that communication that is based on guided noticing, in which speech (typological representations) is linked with images (topological representations) in the form of annotation, leads to better knowledge recall and transfer by the less knowledgeable actor compared with speech-only communication or integrated speech and images without annotation. For our specific research of mobile phone-based communication in the context of less knowledgeable actors, we propose a second set of hypotheses that mobile communication that is based on guided noticing leads to better recall and better transfer of visually represented and verbally represented medical knowledge than speech-only communication or that of integrated speech and images without annotation.

## Methods

### Experimental Design

To test our hypotheses, an intervention study was designed. The experiment was conducted from November 2011 to May 2012 at the University Hospital Basel (Switzerland) with 42 medical students who were obtaining their master’s degrees. All medical students with a master’s curriculum were eligible to participate.

Before starting the experiment, informed written consent was obtained from the participants. In the first step, an experimenter asked the participants to assume the role of an emergency assistant. They briefly analyzed a patient case about a subcapital fracture of the fifth metacarpal bone [51,52] to initiate a consultation with a hand specialist. The patient case included a short text with the initial information about anamnesis and status, as well as a radiological image and photographs showing the limited functional capabilities (impaired fist closure and extension deficit). We chose this case because it is a frequently encountered, yet complex, case for novices. The associated clinical reasoning and treatment involves a great deal of medical and clinical knowledge, including the consideration of radiological, functional, and sociodemographic indicators.

In the second step, the participants were put in communication with a hand surgeon by means of an iPhone 4. To initiate the phone consultation, they were required to briefly characterize the patient case. The specialist provided pertinent standardized advice according to the 3 following experimental conditions/groups: group 1 received verbal explanations (speech only) from the specialist, group 2 received verbal explanations and the radiological image and photographs from the specialist, and group 3 received verbal explanations, the radiological image and the photographs, and annotations (by drawing structures/angle elements) on the radiological image from the specialist (speech, images, and image annotation as guided noticing). Although visual information was varied as described in groups 2 and 3, the verbal information (speech) remained constant in all 3 groups. The case information was prepared by a hand surgeon (UG) and, thereafter, the specialist’s role was assumed by MM.

The technical setting was based on 2 widely available tools: Skype for the communication and Google Drive for the sharing and annotation of the images. Google Drive allows real-time collaboration, inter alia, in the form of sharing and annotating images on desktop and mobile technologies. To avoid variances in the annotations throughout the experiments, the images were preannotated and then displayed in a predefined sequence by the specialist as integral part of the communication setting.

After the conversation with the expert, the participants were asked to complete 2 tasks. Task 1 (knowledge recall) required the participants to write a brief description of the case in which they summarized and justified the relevant points of the conversation (including the diagnosis) in a given time period of 90 seconds (ie, recall of verbally represented knowledge). Second, they were required to draw relevant angles/structures in the radiological image and to estimate the angle size (ie, recall of visually represented knowledge). Task 2 was to measure knowledge transfer. The participants were then given a second case that they had to solve in a similar manner. They were asked to analyze the case information and to develop and justify a diagnosis without specialist support. They wrote a short description of the case including the diagnosis (to determine the transfer of verbally represented knowledge) and drew the relevant angles/structures, and estimated the angle size (to determine the transfer of visually represented knowledge).

In the last step, the participants completed an additional questionnaire about their previous knowledge and experience with fractures. In addition, they answered questions related to the task and experimental conditions. These questions were used as control variables to ensure that there were no differences between the 3 groups.

### Outcome Measures

After the intervention, the participants were requested to estimate the (self-perceived) usefulness of the support provided by the specialist. We also tested the actual recall and transfer of visually and verbally represented medical knowledge. Sample solutions were elaborated by the research team, including medical (hand surgery), and education and psychology experts. For categories and descriptions, see Table 1. The participants’ answers were jointly rated by 2 raters. A negotiated coding approach was applied for the coding procedure because the raters had limited expertise in medical and health sciences and, although they were experienced coders, they were not familiar with applying medical coding schemes. Negotiated approaches have been proposed previously (eg, for transcript analyses when familiarity with a new coding scheme is low) [53]. Negotiated coding was enhanced by resolving disagreements upon discussion with a hand surgeon.

To test the hypotheses with a 2-sided significance level of 5% and a power of 80%, a sample size of 45 participants was calculated, a priori, using G*Power-software [54]. The participants were randomly assigned to 1 of 3 groups using a 3-arm parallel design. A computer-generated list of random numbers was used to distribute the participants according to the principles of simple randomization with a 1:1 ratio.

### Statistical Methods

The data were analyzed using a 3×2 repeated measures ANOVA with a between-subjects factor (group 1, group 2, and group 3) and a within-subjects factor (task 1 and task 2). The effect size (partial η^2^) and the observed power (1–β) for variables that addressed the recall and transfer of verbally and visually represented knowledge were reported. A 1-way ANOVA was calculated for the support offered by the specialist variable. Differences between conditions were assessed using a post hoc comparison. Statistical significance was determined by values *P*<.05 and all analyses were conducted using SPSS version 19 (IBM Corp, Armonk, NY, USA).

### Ethical Considerations

Ethical approval was sought from the regional ethical review board. Because no patients were involved and the participants were considered health care professionals, the board ruled the experiment exempt from further ethical approval and trial registration requirements. Nevertheless, we consulted an expert outside the research team, a professor of ethics at a Swiss university who was part of a separate Swiss ethical board for ethical advice. The confidentiality of the participants was ensured and written informed consent was obtained from every participant before the experiment.

**Table 1:** Measurements of verbally and visually represented knowledge.

Category		Description	Examples
**Recall and transfer of visually represented knowledge**			
	Correctness/completeness of drawn angle elements	Positioning of the following elements: (1) point of intersection, (2) basis line, (3) fracture line, and (4) position of angle	See Figure 1
	Correctness of drawn angle size	Measurement of the angle size drawn by the participant	eg, 75 degrees
	Correctness of estimated angle size	Angle size estimated by the participant	eg, 75 degrees
**Recall and transfer of verbally represented knowledge**			
	Correctness/completeness of functional/radiological characteristics	Measurement of written information, including identification of fracture, type of fracture and position, type of functional limitations (extension deficit and rotation error), and degree of functional restrictions and angulation of the fracture	Fracture of metacarpal V, extension deficit of 10 degrees, and rotation error of 10 degrees, representing 2 functional deficits
	Correctness/completeness of individual sociodemographic patient characteristics	Measurement of written information including age, profession, and dominant hand	Young, employed patient, dominant hand
	Correctness/completeness of overall written answers	Summarizes both measurements	

**Figure 1:**
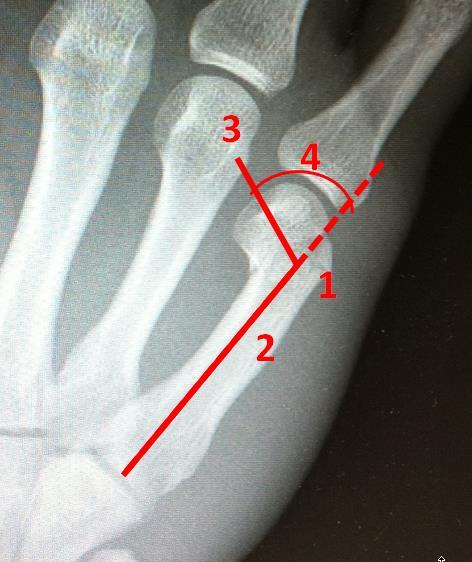
X-ray with fracture as used in the study.

## Results

### Participant Flow

**Figure 2:**
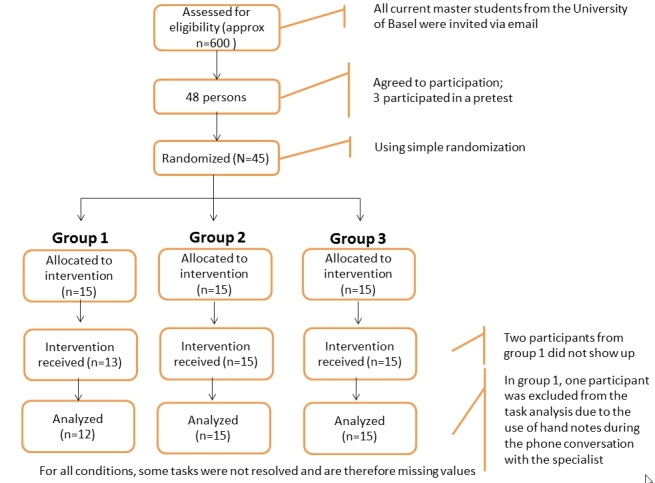
Participant flow diagram.

In all, 48 participants agreed to participate in the experiment. Pretests were conducted with 3 participants to validate the technical feasibility and time limits. The remaining 45 persons were randomized and assigned to the 3 groups. For a flow diagram of participants, see Figure 2.

### Recruitment

Eligible participants were invited by means of email and direct invitation by the medical faculty of the University of Basel in November 2011, using a flyer with basic information. The participants did not receive monetary compensation, but a draw for an iPad was used as an incentive.

### Baseline Demographic Characteristics and Prior Knowledge for Each Group

All 42 participants (23 male, 19 female) were between ages 19 and 38 years (mean 24.57, SD 2.95) and were medical students obtaining their master’s degrees (see Table 2). To determine the bias caused by potentially different levels of prior knowledge in the groups, we asked the participants about their interest in surgical topics (*F*_2,39_=0.10, *P*=.91), their knowledge on the evaluation of fractures (*F*_2,39_=2.17, *P*=.13), prior participation in the treatment of fractures of metacarpal bones (*F*_2,39_=0.96, *P*=.39), and prior experience with diagnosis and treatment of fractures of metacarpal bones (*F*_2,39_=0.58, *P*=.57). No significant differences between the groups were found (for average scores and standard deviation see Table 2).

### Outcomes

#### Support Offered by the Specialist

ANOVA revealed significant differences between group 1 and groups 2 and 3. No significant differences between groups 2 and 3 were observed; a test of between-subjects contrasts yielded the following: *F*_2,39_=6.76, *P*=.003; partial η^2^=0.26 and 1–β=.90. These results suggest that the students in groups 2 and 3 placed significantly more value on the support offered by the specialist. The students’ evaluations of the support offered by the specialist are displayed in Table 3.

**Table 2:** Demographic characteristics and prior knowledge of participants (N=42).

Demographic characteristics		Group 1 (n=12)	Group 2 (n=15)	Group 3 (n=15)
Age, mean (SD)		24.17 (2.12)	25.33 (4.39)	24.13 (1.30)
**Sex, n (%)**				
	Male	7 (17)	8 (19)	8 (19)
	Female	5 (11)	7 (17)	7 (17)
Years of study, mean (SD)		5 (1)	5 (1)	4 (1)
**Prior knowledge, mean (SD)**				
	Experience with touchscreen^a^	4.42 (1.16)	3.53 (1.77)	4.13 (1.50)
	Interest in surgical topics^b^	3.17 (1.19)	3.07 (1.39)	3.27 (1.16)
	Estimated knowledge on the evaluation of fractures (compared to peer students)^b^	2.58 (0.67)	2.20 (1.08)	2.93 (1.03)
	Prior participation in the treatment of fractures of metacarpal bones^b^	1.25 (0.62)	1.13 (0.52)	1.53 (1.13)
	Prior experience in diagnosis and treatment of fractures of metacarpal bones^b^	1.25 (0.87)	1.20 (0.77)	1 (0.00)

^a^Scale: 1=no use; 5=daily use.

^b^Likert scale from 1-5.

**Table 3:** Measurement of specialist support.

Measure	Group, mean (SD)			Mean difference (95% CI)		
	G1 (n=12)	G2 (n=15)	G3 (n=15)	G1–G2	G1–G3	G2–G3
Support offered by the specialist	4.08 (0.90)	4.73 (0.59)	4.93 (0.26)	–0.65^a^ (–1.25, –0.05)	–0.85^a^ (–1.45, –0.25)	–0.20 (–0.76, 0.36)

^a^Mean difference was determined as significant at the *P*<.05 level.

#### Visually Represented Knowledge

We used the following 3 measures to assess the recall and transfer of visually represented medical knowledge: correctness/completeness of drawn angle elements, correctness of drawn angle size, and correctness of estimated angle size (see Table 4). For all these measures, we performed a 2×3 ANOVA with a within-subjects factor (task 1 vs task 2) and a between-subjects factor (group 1 vs group 2 vs group 3). As predicted, for the correctness/completeness of drawn angle elements in both recall and transfer tasks, group 3 (supported with guided noticing) scored significantly higher than the other 2 groups as revealed by the test of between-subjects contrasts (*F*_2,37_=11.32, *P*<.001; partial η^2^=0.38, 1–β=.99). The means, standard deviations, and post hoc contrasts are shown in Table 4.

To test the extent to which the experimental conditions affected the correctness of drawn angle size and the correctness of estimated angle size, we calculated *z* scores and then performed an ANOVA on the standardized data. As hypothesized, group 3 displayed better performance on both measurements: test of between-subjects contrasts relating to the correctness of drawn angle size (*F*_2,37_=8.81, *P*<.001; partial η^2^=0.32, 1–β=.96) and the test of between-subjects contrasts relating to the correctness of estimated angle size (*F*_2,37_=7.67, *P*<.001; partial η^2^=0.30, 1–β=.93). More precisely, for knowledge recall, post hoc tests showed that the ability to draw correct angle sizes and the ability to estimate the size of the drawn angle was significantly higher for group 3 when compared with the other 2 groups. However, a similar result was not supported in the transfer task, in which significant differences were observed only between groups 1 and 3.

**Table 4:** Recall and transfer of visually represented medical knowledge.

Submeasures of visually represented medical knowledge		Group, mean (SD)			Adjusted difference, mean (95% CI)		
		G1 (n=11)	G2 (n=15)	G3 (n=14)	G1–G2	G1–G3	G2–G3
**Correctness/completeness of drawn angle elements**							
	Task 1: Recall	1.23 (1.54)	1.83 (1.21)	3.29 (1.14)	–0.61 (–1.88, 0.67)	–2.06^a^ (–3.35, –0.76)	–1.45^a^ (–2.65, –0.26)
	Task 2: Transfer	1.82 (1.52)	2.03 (1.32)	3.57 (0.47)	–0.22 (–1.38, 0.95)	–1.75^a^ (–2.93, –0.58)	–1.53^a^(–2.62, –0.45)
**Correctness of drawn angle size** ^b^							
	Task 1: Recall	0.54 (.91)	0.36 (.95)	–0.78 (0.63)	0.18 (–0.66, 1.01)	1.32^a^ (0.47, 2.17)	1.14^a^ (0.36, 1.92)
	Task 2: Transfer	0.48 (0.98)	0.15 (1.01)	–0.56 (0.76)	0.33 (–0.59, 1.25)	1.05^a^ (0.11, 1.98)	0.71 (–0.15, 1.57)
**Correctness of estimated angle size** ^b^							
	Task 1: Recall	0.51 (0.92)	0.37 (0.96)	–0.74 (0.69)	0.14 (–0.73, 1.02)	1.25^a^ (0.38, 2.12)	1.11^a^ (0.29, 1.93)
	Task 2: Transfer	0.45 (0.98)	0.15 (1.05)	–0.55 (0.75)	0.30 (–0.65, 1.25)	1.00^a^ (0.06, 1.95)	0.70 (–0.18, 1.59)

^a^The mean difference was determined to be significant (*P*<.05).

^b^Small numbers indicate better performance.

#### Verbally Represented Knowledge

We used the following 2 submeasures to assess the recall and transfer of verbally represented medical knowledge: the correctness/completeness of functional/radiological characteristics and the correctness/completeness of individual sociodemographic patient characteristics (see Table 2). Statistical analysis was performed on the general measure of correctness and completeness of overall written answers (Table 5). Scores that assessed the recall and transfer of verbally represented knowledge were analyzed by means of a 2×3 repeated ANOVA with a within-subjects factor (task 1 vs task 2) and a between-subjects factor (group 1 vs group 2 vs group 3). The results showed no significant differences between the 3 groups, with respect to the correctness and completeness of verbally represented knowledge for both the recall and transfer tasks. The calculated tests of between-subjects effects (factor 1) did not reach statistical significance with a level of *P*<.05 (*F*_2,39_=0.58, *P*=.58; partial η^2^=0.03, 1–β=.13).

**Table 5:** Correctness/completeness of overall written answers for recall and transfer.

Measure	Group, mean (SD)			Mean difference (95% CI)		
	G1 (n=12)	G2 (n=15)	G3 (n=15)	G1–G2	G1–G3	G2–G3
Task 1: Recall	5.92 (2.07)	6.33 (2.02)	6.67 (2.58)	–0.42 (–2.60, 1.76)	–0.75 (–2.93, 1.43)	–0.33 (–2.39, 1.72)
Task 2: Transfer	3.00 (2.34)	3.00 (2.54)	3.67 (2.55)	0.00 (–2.41, 2.41)	–0.67(–3.08, 1.74)	–0.67 (–2.94, 1.61)

#### Performance Between Task 1 and 2

With respect to verbally represented knowledge (correctness/completeness of overall written answers), we observed significant differences in participants’ performance between task 1 and 2 using a test of within-subjects effects (*F*_1,39_=43.72, *P*<.001; partial η^2^= 0.53, 1–β=1), whereby the performance decreased in task 2, independent of the experimental condition (task 1: mean 6.30, SD 0.39; task 2: mean 3.22, SD 0.38; adjusted difference: mean 3.08, 95% CI 2.14-4.03). We observed no significant differences with respect to visually represented knowledge relating to the correctness/completeness of drawn angle elements (*F*_1,37_=3.37, *P*=.06); the correctness of drawn angle (*F*_2,37_= 0.02, *P*=.89); and the correctness of estimated angle (*F*_1,36_=0.04, *P*=.84). We observed a tendency toward improved performance when assessing the variable correctness/completeness of drawn angle elements in task 2 (see Table 6).

**Table 6:** Visually represented knowledge: performance between tasks 1 (knowledge recall) and 2 (knowledge transfer).

Measure	Task, mean (SD)		Mean difference (G1–G2) (95% CI)
	Task 1: Recall	Task 2: Transfer	
Correctness/completeness of drawn angle elements	2.17 (1.51)	2.51 (1.38)	–0.36 (–0.73, 0.07)
Correctness of drawn angle size^a^	0.01 (1.01)	–0.01 (1.00)	0.20 (–0.27, 0.31)
Correctness of estimated angle size^a^	0.01 (1.01)	–0.01 (1.00)	0.30 (–0.26, 0.32)

^a^Small numbers indicate better performance.

## Discussion

### Principal Results

In light of the rapid adoption of mobile technology, such as smartphones, for clinical communication, this study systematically compares different synchronous mobile phone-based communication modes, including (1) speech only, (2) speech and images, and (3) speech, images, and image annotation (guided noticing). Using an experimental approach, we investigated to what extent these modes affect knowledge processes in clinical communication as indicated by (1) recall and (2) transfer of visually and verbally represented knowledge by a less knowledgeable medical actor. Our variations and hypotheses were informed by psychological theories from related research in the cognitive and sociocognitive sciences.

In the conditions where speech was integrated with images (groups 2 and 3), participants evaluated the support provided by the specialist as significantly more positive compared to the speech-only condition (group 1). However, in measuring actual knowledge gains, significant positive effects on the recall and transfer of visually represented medical knowledge were only measured in group 3, which integrated speech, images, and image annotation (guided noticing). With respect to verbally represented medical knowledge, no significant differences were measured. In other words, the presentation of visual information did not contribute to the recall and retention of verbally represented knowledge.

These findings contribute original data to at least 2 broad discourses: (1) the practice of clinical communication and medical informatics, and (2) psychological, cognitive, and sociocognitive learning and communication theories.

### Implications for Medical/Clinical Communication and Medical Informatics

Unlike most of the other studies that have investigated the contributions of mobile technologies to clinical communication and knowledge exchange, we used a randomized controlled experimental design. The findings suggest that, despite increased self-perceived effectiveness of the use of visual communication modes (compared to a speech-only mode), the understanding and knowledge gains of less knowledgeable medical actors are only enhanced if annotations are used to integrate speech with images. The finding that the actual effectiveness of mobile multimodal communication is different than the self-perceived effectiveness is critical and may negatively affect patient treatment, such as during a specialist consultation; if a requesting doctor were to deem phone-based advice from the specialist (without annotation) as sufficient, this could result in poor decision making with regard to the requesting doctor’s further treatment of the patient. In addition, the present results challenge studies in which positive outcomes of knowledge gains of rich smartphone-based consultations were determined using only self-perceived evaluation (eg, [35]). The present study also highlights the value of annotation for clinical communication and medical informatics. Hospital management may consider this mode of communication when developing or buying new communication solutions, including mobile solutions. Senior clinicians may learn from our findings that less knowledgeable actors, students, and younger colleagues are much more likely to benefit from explanations that integrate speech and visual information by means of labeling and annotating, which is a practice that should not be limited to mobile communication. Recently, it was shown how doctors who create rich multimodal representations by using gestures to connect speech with different computational and bodily representations can improve the understanding of less knowledgeable colleagues [55]. These aspects of communicating, which are highly relevant for clinical communication and learning, are widely neglected in the current clinical practice and should be more thoroughly considered in the future.

### Theoretical Implications and Discussion

To our knowledge, our experimental study is the first to examine the dual channel and multimedia theories in applied, complex, and authentic situations, such as clinical communication. Our first set of hypotheses (that mobile communication involving integrated speech and images leads to better recall and better transfer of visually represented and verbally represented medical knowledge than the speech-only condition) was based on the dual channel and multimedia theories: we assumed that synchronous mobile communication which integrated images and speech (group 2; ie, contingent multimedia information) would enhance a less knowledgeable actor’s understanding of the subject matter compared with the speech-only condition (group 1). However, we found that using images in group 2 did not lead to significantly improved learning compared to the speech-only condition (group 1). This result challenges other studies from the related field of cognitive research (eg, [56]). A possible explanation lies in our specific experimental clinical setting. During the preparation phase, the images were shown to all participants (including those in the first condition) to initiate the specialist consultation. It is possible that the participants from the speech-only group could later recall these images during the communication phase, thus compensating to some extent for not having the real images available. In other words, the participants in the speech-only condition might have invested more mental effort by imagining the images.

Our second set of hypotheses (that mobile communication based on guided noticing leads to better recall and better transfer of visually represented and verbally represented medical knowledge than speech-only communication or that of integrated speech and images without annotation) was derived from the concept of guided noticing [40]. We assumed that speech, images, and annotations (guided noticing) in group 3 would lead to knowledge gains with respect to the recall and transfer of both verbally and visually represented medical knowledge (compared with the other conditions). Our results confirm an increased ability to recall visually represented medical knowledge in the guided noticing condition for all 3 submeasures when compared with the other 2 conditions. With regard to the knowledge transfer, the correctness/completeness submeasure of drawn angle elements was significantly affected compared with the other conditions. However, the correctness of the drawn and estimated angle was significantly superior in group 3 (guided noticing) only compared to group 1 (the speech-only condition) and not when compared to group 2 (speech and images).

Taken together, these results might be readily explained by the fact that the correctness/completeness of drawn angle elements submeasure (see Figure 1) requires a complex and comprehensive understanding of the image and knowledge that cannot be compensated for over time or by increased mental effort with imagination. With this difference in mind, the results demonstrate the importance of guided noticing as a concept and we recommend this mode of communication for the clinical practice.

In summary, the simple integration of images and speech in mobile phone-based communication did not lead to significantly improved knowledge gains (compared with speech-only communication). Our results confirmed the value of guided noticing in communication. Moreover, they support the view that guided noticing is particularly important in situations involving complex visual subject matter, typical in clinical communication.

### Strengths, Limitations, Generalizability, and Future Research

In addition to the strengths indicated (ie, using an experimental randomized design in authentic and complex settings), our study is based on firm theoretical underpinnings. Our design draws on and is discussed in terms of a broad theoretical spectrum, combining both cognitive (dual channel/multimedia theory) and sociocognitive (guided noticing) perspectives. This is an approach we deem suitable to address the complexities of clinical communication. However, the findings need to be interpreted and generalized with respect to several limitations. First, we recruited medical students as participants. This is a limitation because students represent a specific group of clinical actors. Although they have accumulated some clinical experiences in the course of their education, they represent a group with limited medical expertise and experience. Second, we tested the hypotheses exclusively against a specific clinical case, a specialist consultation. From a practical viewpoint, our study was conducted in a laboratory setting in a hospital (ie, in an environment that was relatively stable compared to the hectic, noisy, interrupted, and chaotic contexts of real clinical communication), where learning and teaching additionally depend on the nonlinear interplay of a variety of different factors [57,58]. In real clinical settings, it remains unclear, for example, to what extent clinicians are willing to use annotations and extended phone-based explanations. Similarly, to what extent an additional device (eg, a mobile phone) may contribute to cognitive overload in an environment where multiple sources already claim the full attention of a clinician needs to be explored [59]. A further limitation of our experimental design is that prior knowledge was not evaluated in the form of a test, but only as a self-reported measurement. It also needs to be acknowledged that the negotiated coding may represent a limitation because the widely recognized Guidelines for Reporting Reliability and Agreement Studies (GRRAS) recommend using the mean of 2 or more raters to increase reliability [60]. Although we fully agree with GRRAS, in our case we deem the negotiated coding approach a legitimate technique, as recently proposed in the research literature [53]. Moreover, negotiated coding was strengthened by resolving disagreement and uncertainty with a hand surgeon.

In conclusion, the present study yields interesting insights into the knowledge-based effects of multimodal, phone-based communication, but it cannot provide definite accounts of all the observed phenomena. Future research addressing these questions may (1) use different user groups (eg, residents), (2) use different case representations (eg, MRI), (3) test the system not only in authentic but in real clinical settings (eg, by evaluating prior and post hoc knowledge), and (4) capture knowledge-based effects over a longer period of time.

## Multimedia Appendix 1

CONSORT-EHEALTH checklist V1.6.2 [61].

## Abbreviations

CT: computed tomography
CTA: computed tomography angiography
GRRAS: Guidelines for Reporting Reliability and Agreement Studies
NCCT: noncontrast computed tomography
