# Supplementary material for: Smartphones as Multimodal Communication Devices to Facilitate Clinical Knowledge Processes: Randomized Controlled Trial
Source: J Med Internet Res. 2013 Nov 27;15(11):e263. doi: 10.2196/jmir.2758 (PMC3868983; doi:10.2196/jmir.2758)
Supplement: Supplementary file 1 [file jmir_v15i11e263_app1.pdf]

CONSORT-EHEALTH Checklist V1.6.2 Report

(based on CONSORT-EHEALTH V1.6), available at [http://tinyurl.com/consort-ehealth-v1-6].

2758

Date completed

9/17/2013 9:05:06

by

Pimmer Christoph

Smartphones as multimodal communication devices to facilitate clinical knowledge processes - a randomized controlled trial

TITLE

1a-i) Identify the mode of delivery in the title

Yes: the smartphone is specified as the mode of delivery

1a-ii) Non-web-based components or important co-interventions in title

Yes: the smartphone is specified as the mode of delivery

1a-iii) Primary condition or target group in the title

Yes: the title indicates that the paper refers to clinicians/medical actors

ABSTRACT

1b-i) Key features/functionalities/components of the intervention and comparator in the METHODS section of the ABSTRACT

"First, all participants analyzed a standardized case, a patient with a subcapital fracture of the fifth metacarpal bone, based on a radiological image, photographs of the hand, and textual descriptions. These participants were asked to consult a remote surgical specialist via a smartphone. In doing so, they were randomly assigned to three experimental conditions/groups. In group 1 the specialist provided verbal explanations to the participants (speech only); in group 2, in addition to verbal explanations, the specialist displayed the radiological image and the photographs to the participants. In group 3, the specialist provided verbal explanations, displayed the radiological image and the photographs, and annotated the radiological image by drawing structures/angle-elements (speech, images and image annotation."

1b-ii) Level of human involvement in the METHODS section of the ABSTRACT

Human involvement: "first, all participants analyzed a standardized case .. " " the specialist provided verbal explanations … "

1b-iii) Open vs. closed, web-based (self-assessment) vs. face-to-face assessments in the METHODS section of the ABSTRACT

"The experiment was conducted from November 2011 to May 2012 at the University Hospital Basel (Switzerland) with 42 medical students who were obtaining their master's degrees. Eligible participants were recruited using e-mail and direct invitation by the medical faculty of the University of Basel.. […] "After the consultation, as a knowledge recall measurement, the participants were asked to write brief summaries of the case (verbally represented knowledge) and to re-analyze the diagnostic images (visually represented knowledge). To assess knowledge transfer, the participants analyzed a new, similar case without specialist support."

1b-iv) RESULTS section in abstract must contain use data

"The experiment was conducted ... with 42 medical students who were obtaining their master's degrees. ... In doing so, they were randomly assigned to three experimental conditions/groups."

1b-v) CONCLUSIONS/DISCUSSION in abstract for negative trials

not applicable since trial was not negative

INTRODUCTION

2a-i) Problem and the type of system/solution

The intervention is based on (1) inter-clinician communication and mobile phones, (2) using rich communication modes such as speech, images and annotation, with a specific focus on (3) knowledge exchange. For all of thre three themes an introductory/background section is provided.

2a-ii) Scientific background, rationale: What is known about the (type of) system

Please see the points specified above

METHODS

3a) CONSORT: Description of trial design (such as parallel, factorial) including allocation ratio

All hypotheses are thoroughly delineated from two different theoretical strands: (1) the dual coding and multimedia learning theorie; (2), the second set relates to the notion of guided noticing in communication:

"In order to address this research gap, we delineated two sets of hypotheses based on a cognitive science approach. The first set of hypotheses relates to theories of dual coding and multimedia learning [36-39], the second set relates to the notion of guided noticing in communication [40, 41]. Concerning cognitive science, it has been shown that images support human information processing and understanding. This is not only because images convey different information than words (concrete vs. abstract), but because text and images are processed in two different "channels" or "modes" [36-39]. The dual channel processing assumption asserts that humans have separate channels for processing words and images and that they use them both to construct coherent mental knowledge representations [36, 38, 39]. Empirical evidence has demonstrated picture superiority effects, i.e., that people can recall information better from both words and images than from words alone [37, 42-44]. Moreover, a vast body of research on learning with multimedia [37, 43] suggests that when words (written words/speech) and images are presented together and in a well-designed and integrated manner (e.g., redundant and spatially contingent, among others), they support the construction of coherent mental representations, and, thus, learning, (e.g., learning about the functioning of physical systems) [45]. This idea is especially true for less knowledgeable learners [e.g. 46] and in situations where test items were presented in image-based form [47]. However, research on dual coding and multimedia learning was mainly conducted in experimental studies using simple content. There is a paucity of evidence that demonstrates learning effects in applied and more complex situations, such as that of inter-clinician communication and knowledge exchange. According to multimedia theory, we formulated the first assumption for the experimental design of mobile phone-based medical communication:
Mobile phone-based medical communication, where speech and images are combined to enhance the understanding of complex subject matter, leads to better understanding by a less knowledgeable actor when compared to the speech-only condition. Therefore, knowledge recall and transfer of verbally and visually represented knowledge should increase when compared to the speech-only condition.
Therefore, in our specific case of mobile communication and less knowledgeable actors, we hypothesize the following:
H1a: Mobile communication, involving integrated speech and images, leads to better recall of visually represented medical knowledge than the speech-only condition.
H1b: Mobile communication, involving integrated speech and images, leads to better transfer of visually represented medical knowledge than the speech-only condition.
H1c: Mobile communication, involving integrated speech and images, leads to better recall of verbally represented medical knowledge than the speech-only condition.
H1d: Mobile communication, involving integrated speech and images, leads to better transfer of verbally represented medical knowledge than the speech-only condition.
Concerning the second set of hypotheses (H2) we derive our assumptions from the concept of guided noticing. Guided noticing describes how specific digital technology affordances can be used in distributed collaborative settings to direct joint attention to notice specific elements in visual media (e.g., videos, images). Guided noticing involves highlighting (e.g., annotation), naming and commenting and thus allows the assignment of typological representations (culturally meaningful categories) to topological representations (e.g., images). This is especially important when experts interpret visualizations in detail and explain their interpretations to others. Here, guided noticing can be a way to establish "common ground" [48] in communication between professionals (e.g., in educational settings), for example by using annotations. The creation of annotations is considered as a grounding or communication act by users (rather than designers), who intend to close gaps in common ground required to complete a task [32]. Guided noticing can also serve to develop domain expertise and a "professional vision" [49], e.g., the training of diagnostic skills with x-rays [41]. Professional vision was empirically investigated, and characterized by Goodwin [49] as "… socially organized ways of seeing and understanding events that are answerable to the distinctive interests of a particular social group" (p. 606). Case studies of professional practices (such as developing coding schemes, or highlighting) with domain experts (e.g., archeologists in field research, lawyers in courtroom) were conducted analyzing these practices in great detail. Yet, empirical research on guided noticing [41] is still sparse. In our own previous research on history learning with advanced video tools, we identified episodes of guided noticing and analyzed them in relation to the successful acquisition of visual knowledge [50]. Our present study extends the existing qualitative research by adding original quantitative data on the effects of highlighting (by annotation) for guided noticing in the specific area of medical knowledge."

Accordingly, we formulated the following assumption:
Communication that is based on guided noticing, where speech (typological representations) is linked with images (topological representations) in the form of annotation, leads to better knowledge recall and transfer by the less knowledgeable actor compared with speech-only communication or integrated speech and images without annotation. For our specific research of mobile phone-based communication in the context of less knowledgeable actors, we propose the second set of the following hypotheses:

H2a: Mobile communication that is based on guided noticing leads to better recall of visually represented medical knowledge than speech-only communication or that of integrated speech and images without annotation.
H2b: Mobile communication that is based on guided noticing leads to better transfer of visually represented medical knowledge than speech-only communication or that of integrated speech and images without annotation.
H2c: Mobile communication that is based on guided noticing leads to better recall of verbally represented medical than speech-only communication or that of integrated speech and images without annotation.
H2d: Mobile communication that is based on guided noticing leads to better transfer of verbally represented medical knowledge than speech-only communication or that of integrated speech and images without annotation.

3b) CONSORT: Important changes to methods after trial commencement (such as eligibility criteria), with reasons
No changes after trial commencement
3b-i) Bug fixes, Downtimes, Content Changes
No bug fixes or unexpected events occurred during the experiment, except for one participant who took notes during the conversation and was subsequently excluded, as specified in the flow diagram in Figure 2.
4a) CONSORT: Eligibility criteria for participants
"All medical students with a master's curriculum were eligible to participate."
4a-i) Computer / Internet literacy
To address literacy we measured the participants' experiences with touchscreens; no significant difference were found as reported in Table 3.
4a-ii) Open vs. closed, web-based vs. face-to-face assessments:
"Eligible participants were invited by means of e-mail and direct invitation by the medical faculty of the University of Basel in November 2011, using a flyer with basic information."
4a-iii) Information giving during recruitment
"Before starting the experiment, informed written consent was obtained from the participants. "
"Eligible participants were invited by means of e-mail and direct invitation by the medical faculty of the University of Basel in November 2011, using a flyer with basic information. The participants did not receive monetary compensation but took part in a gift lottery with an iPad as an incentive."
4b) CONSORT: Settings and locations where the data were collected

"The single-center experiment was conducted from November 2011 to May 2012 in a meeting room at the University Hospital Basel in Switzerland."

4b-i) Report if outcomes were (self-)assessed through online questionnaires

Not applicable since no web-based self-assessment was used.

4b-ii) Report how institutional affiliations are displayed

Not applicable, no bias.

5) CONSORT: Describe the interventions for each group with sufficient details to allow replication, including how and when they were actually administered

5-i) Mention names, credential, affiliations of the developers, sponsors, and owners

Not applicable as we used publically available software and hardware as specified:
"The technical setting was based on two widely available tools: Skype™ for the communication; and Google Drive™, for the sharing and annotation of the images. Google Drive™ allows real-time collaboration, inter alia, in the form of sharing and annotating images on Desktop and mobile technologies. In order to avoid variances in the annotations throughout the experiments, the images were pre-annotated and then displayed in a pre-defined sequence by the specialist as integral part of the communication setting. "

5-ii) Describe the history/development process

Not applicable since no development was involved

5-iii) Revisions and updating

Not applicable since no development was involved

5-iv) Quality assurance methods

Not applicable since no development was involved

5-v) Ensure replicability by publishing the source code, and/or providing screenshots/screen-capture video, and/or providing flowcharts of the algorithms used

Not applicable since no development was involved and the tools are publically available.

5-vi) Digital preservation

Not applicable since no development was involved and the tools are publically available.

5-vii) Access

"In the second step, the participants were put in communication with a hand surgeon by means of an iPhone 4.
...
In group 1 the specialist provided verbal explanations to the participants (speech only); in group 2, in addition to verbal explanations, ...
....
The participants did not receive monetary compensation but took part in a gift lottery with an iPad as an incentive. "

5-viii) Mode of delivery, features/functionalities/components of the intervention and comparator, and the theoretical framework

"In the second step, the participants were put in communication with a hand surgeon by means of an iPhone 4. To initiate the phone consultation, they were required to briefly characterize the patient case. The specialist provided pertinent standardized advice according to the 3 following experimental conditions/groups:
In group 1 the specialist provided verbal explanations to the participants (speech only); in group 2, in addition to verbal explanations, the specialist displayed the radiological image and the photographs to the participants. In group 3, the specialist provided verbal explanations, displayed the radiological image and the photographs, and annotated the radiological image by drawing structures/angle-elements (speech, images and image annotation as guided noticing). While visual information was varied as described in groups 2 and 3, the verbal information (speech) remained constant in all three groups. The case information was prepared by a hand surgeon (UG), and thereafter, the specialist's role was assumed by MM.
The technical setting was based on two widely available tools: Skype™ for the communication; and Google Drive™, for the sharing and annotation of the images. Google Drive™ allows real-time collaboration, inter alia, in the form of sharing and annotating images on Desktop and mobile technologies. In order to avoid variances in the annotations throughout the experiments, the images were pre-annotated and then displayed in a pre-defined sequence by the specialist as integral part of the communication setting. "

5-ix) Describe use parameters

Not applicable. For communication modes please see the group-specific description above.

5-x) Clarify the level of human involvement

Two persons were involved in the experimental setting in addition to the participant. This is clearly described:

"In the first step, an experimenter asked the participants to assume the role of an emergency assistant. "

"In the second step, the participants were put in communication with a hand surgeon by means of an iPhone 4."

5-xi) Report any prompts/reminders used

Not applicable. Two experimenters guided the participant as indicated.

5-xii) Describe any co-interventions (incl. training/support)

Not applicable; no co-interventions used.

6a) CONSORT: Completely defined pre-specified primary and secondary outcome measures, including how and when they were assessed

Outcome measures are explained as follows:

"After the intervention, the participants were requested to estimate the (self-perceived) usefulness of the support provided by the specialist. We also tested the actual recall and transfer of visually and verbally represented medical knowledge. Sample solutions were elaborated by the research team, including medical (hand surgery), as well as education and psychology experts. For categories and descriptions, see Table 2. "

6a-i) Online questionnaires: describe if they were validated for online use and apply CHERRIES items to describe how the questionnaires were designed/deployed

Not applicable since no online questionnaires were used

6a-ii) Describe whether and how "use" (including intensity of use/dosage) was defined/measured/monitored

See 6a)

6a-iii) Describe whether, how, and when qualitative feedback from participants was obtained

No qualitative feedback was gathered.

6b) CONSORT: Any changes to trial outcomes after the trial commenced, with reasons

No changes commenced

7a) CONSORT: How sample size was determined

7a-i) Describe whether and how expected attrition was taken into account when calculating the sample size

To test the hypotheses with a two-sided significance level of 5% and a power of 80 percent, a sample size of 45 participants was calculated, a priori, using G*Power-software (Baguley, 2004). Baguley, T. (2004). Understanding statistical power in the context of applied research. Applied Ergonomics, 35(2), 73-80. doi: 10.1016/j.apergo.2004.01.002

7b) CONSORT: When applicable, explanation of any interim analyses and stopping guidelines

Not applicable since no interim analysis was conducted and no stopping guidelines were defined.

8a) CONSORT: Method used to generate the random allocation sequence

The participants that were randomly assigned to one of three groups using a three-arm parallel design. A computer-generated list of random numbers was used to distribute the participants according to the principles of simple randomization with a 1:1 ratio.

8b) CONSORT: Type of randomisation; details of any restriction (such as blocking and block size)

No restrictions. For procedure see please see above. (8a)

9) CONSORT: Mechanism used to implement the random allocation sequence (such as sequentially numbered containers), describing any steps taken to conceal the sequence until interventions were assigned

"A computer-generated list of random numbers was used to distribute the participants according to the principles of simple randomization with a 1:1 ratio."

10) CONSORT: Who generated the random allocation sequence, who enrolled participants, and who assigned participants to interventions

This was done by the researchers in the following format: "a computer-generated list of random numbers was used to distribute the participants according to the principles of simple randomization with a 1:1 ratio. "

11a) CONSORT: Blinding - If done, who was blinded after assignment to interventions (for example, participants, care providers, those assessing outcomes) and how

11a-i) Specify who was blinded, and who wasn't

The research design with three different communication modes did not require any form of participant blinding.

11a-ii) Discuss e.g., whether participants knew which intervention was the "intervention of interest" and which one was the "comparator"

Not applicable since three different communication designs were used and all of them were of interest.

11b) CONSORT: If relevant, description of the similarity of interventions

Not applicable

12a) CONSORT: Statistical methods used to compare groups for primary and secondary outcomes

"The data were analyzed using a 3x2 repeated measures analysis of variance (ANOVA) analysis of variance (ANOVA) with a between-subjects factor (group 1, group 2 and group 3) and a within-subjects factor (task 1 and task 2). The effect size (2, partial eta-squared) and the observed power (1-) for variables that addressed the recall and transfer of verbally and visually represented knowledge were reported. A one-way ANOVA was calculated for the variable "support offered by the specialist." Differences between conditions were assessed using a post hoc comparison. Statistical significance was determined by values, p<.05, and all analyses were conducted using SPSS version 19 (SPSS Inc., an IBM Company). "

12a-i) Imputation techniques to deal with attrition / missing values

Not applicable since no imputation techniques were used. Lacking data regarded as "missing values".

12b) CONSORT: Methods for additional analyses, such as subgroup analyses and adjusted analyses

Not applicable since no additional analyses wered conducted

RESULTS

13a) CONSORT: For each group, the numbers of participants who were randomly assigned, received intended treatment, and were analysed for the primary outcome

"Forty-eight participants agreed to participate in the experiment. Pre-tests were conducted with 3 participants to validate the technical feasibility and time limits. The remaining 45 persons were randomized and assigned to three groups; for a flow diagram, see Figure 2."

13b) CONSORT: For each group, losses and exclusions after randomisation, together with reasons

Please see the CONSORT flow diagram (Figure 2)

13b-i) Attrition diagram

Information about losses is integrated in the CONSORT flow diagram (Figure 2)

14a) CONSORT: Dates defining the periods of recruitment and follow-up

Eligible participants were invited by means of e-mail and direct invitation by the medical faculty of the University of Basel in November 2011, using a flyer with basic information.

14a-i) Indicate if critical "secular events" fell into the study period

Not applicable

14b) CONSORT: Why the trial ended or was stopped (early)

Trial was not stopped early

15) CONSORT: A table showing baseline demographic and clinical characteristics for each group

"Table 3 Demographic characteristics and prior knowledge" provides demographic characteristics.

15-i) Report demographics associated with digital divide issues

Pleas see "Table 3 Demographic characteristics and prior knowledge", item "Experience with touchscreen"

16a) CONSORT: For each group, number of participants (denominator) included in each analysis and whether the analysis was by original assigned groups

16-i) Report multiple "denominators" and provide definitions

N's and effect sizes are indicated in table 4, 5 and 6

16-ii) Primary analysis should be intent-to-treat

Not applicable

17a) CONSORT: For each primary and secondary outcome, results for each group, and the estimated effect size and its precision (such as 95% confidence interval)

"ANOVA revealed significant differences between group 1 and groups 2 and 3. No significant differences between groups 2 and 3 were observed; a test of between-subjects contrasts yielded the following: F(2, 39)=6.76, p=.003; 2=0.26 and 1-=.90. These results suggested that the students in groups 2 and 3 placed significantly more value on the support offered by the specialist.
…
As predicted, for the correctness/completeness of drawn angle elements in both recall and transfer tasks, group 3 (supported with guided noticing) scored significantly higher than the other two groups, as revealed by the test of between-subjects contrasts, F(2, 37)=11.32, p=.00; 2 =.38, 1-=.99. The means, standard deviations and post hoc contrasts are shown in Table 5.
To test the extent to which the experimental conditions affected the correctness of drawn angle size and the correctness of estimated angle size, we calculated Z-scores and then performed an ANOVA on the standardized data. As hypothesized, group 3 displayed better performance on both measurements: test of between-subjects contrasts relating to the correctness of drawn angle size, F(2, 37)=8.81, p=.00; 2 =.32, 1-=.96; and test of between-subjects contrasts relating to the correctness of estimated angle size, F (2, 37)=7.67, p=.00; 2=.30, 1-=.93.
…
The results showed no significant differences between the three groups, with respect to the correctness and completeness of verbally represented knowledge for both the recall and transfer tasks. The calculated tests of between-subjects effects (factor 1) did not reach statistical significance with a level of .05 (F(2, 39)=.58, p=.58; 2 = .03, 1-=.13). The presentation of visual information did not contribute to the recall and retention of verbally represented knowledge. None of the hypotheses referring to verbally represented knowledge (H1c, H2c, H1d, H2d) were confirmed. "

The rest of the data including confidence intervals can be found in table 4,5 and 6
17a-i) Presentation of process outcomes such as metrics of use and intensity of use
No process outcomes defined and measured
17b) CONSORT: For binary outcomes, presentation of both absolute and relative effect sizes is recommended
No binary outcomes were measured
18) CONSORT: Results of any other analyses performed, including subgroup analyses and adjusted analyses, distinguishing pre-specified from exploratory
Not applicable since no other analysis was performed
18-i) Subgroup analysis of comparing only users
Not applicable since no subgroup analysis was performed.
19) CONSORT: All important harms or unintended effects in each group
No harms and unintended effects occurred
19-i) Include privacy breaches, technical problems
No occurences
19-ii) Include qualitative feedback from participants or observations from staff/researchers
No qualitative feedback provided/measured

DISCUSSION

20) CONSORT: Trial limitations, addressing sources of potential bias, imprecision, multiplicity of analyses
20-i) Typical limitations in ehealth trials
"A further limitation of our experimental design is that prior knowledge was not evaluated in the form of a test but only as self-reported measurement. It also needs to be acknowledged that the negotiated coding may represent a limitation as the widely recognized guidelines for "Reporting Reliability and Agreement Studies" (GRRAS) recommend using the mean of two or more raters to increase reliability [60]. While, we fully agree with GGRAS, in our case we deem the negotiated coding approach a legitimate technique, as recently proposed in the research literature[53]; Moreover, negotiated coding was strengthened by resolving disagreement and uncertainty with a hand surgeon. "
21) CONSORT: Generalisability (external validity, applicability) of the trial findings
21-i) Generalizability to other populations
"However, the findings need to be interpreted and generalized with respect to several limitations. First, we recruited medical students as participants. This is a limitation because students represent one specific group of clinical actors. While they have accumulated some clinical experiences in the course of their education, they represent a group with limited medical expertise and experience. Second, we tested the hypotheses exclusively against one specific clinical case, a specialist consultation. From a practical viewpoint, our study was conducted in a laboratory setting in a hospital, i.e., in an environment that was relatively stable compared to the hectic, noisy, interrupted and chaotic contexts of real clinical communication, where learning and teaching additionally depend on the non-linear interplay of a variety of different factors [57, 58]. In real clinical settings, it remains unclear, for example, to what extent clinicians are willing to use annotations and extended phone-based explanations. Similarly, to what extent an additional device, e.g., a mobile phone, may contribute to cognitive overload in an environment where multiple sources already claim the full attention of a clinician needs to be explored [59]."

21-ii) Discuss if there were elements in the RCT that would be different in a routine application setting
See comment above
22) CONSORT: Interpretation consistent with results, balancing benefits and harms, and considering other relevant evidence
22-i) Restate study questions and summarize the answers suggested by the data, starting with primary outcomes and process outcomes (use)
" Principal results
In light of the rapid adoption of smartphones for clinical communication, this study systematically compares different synchronous mobile phone-based communication modes including (1) speech-only, (2) speech and images and (3) speech, images and image annotation (guided noticing). Using an experimental approach, we investigated to what extent these modes impact knowledge processes in clinical communication as indicated by (a) recall and (b) transfer of visually and verbally represented knowledge by a less knowledgeable medical actor. Our variations and hypotheses were informed by psychological theories from related research in the cognitive and socio-cognitive sciences. "
22-ii) Highlight unanswered new questions, suggest future research
"Future research addressing these questions may (a) use different user groups (e.g., residents), (b) different case representations (e.g., MRI), (c) test the system not only in authentic but real clinical settings (by prior and post hoc knowledge and evaluation) and (d) capture knowledge-based effects over a longer period of time. "

Other information

23) CONSORT: Registration number and name of trial registry
Ethical approval was sought from the regional ethical review board. Since no patients were involved and since the participants were considered as healthcare professionals, the board ruled the experiment exempt from further ethical approval and trial registration requirements.
24) CONSORT: Where the full trial protocol can be accessed, if available
Available on request from authors

25) CONSORT: Sources of funding and other support (such as supply of drugs), role of funders

"The authors also thank the project sponsors and partners, the CTI - the Swiss Confederation's Innovation Promotion Agency, AMTS, Agfa Healthcare, University Hospital Basel, Hightech Research Center of Cranio-Maxillofacial Surgery University of Basel and the University of Applied Sciences Northwestern Switzerland for their support of this work. "

X26-i) Comment on ethics committee approval

"Ethical approval was sought from the regional ethical review board. Since no patients were involved and since the participants were considered as healthcare professionals, the board ruled the experiment exempt from further ethical approval and trial registration requirements. Nevertheless we consulted an expert outside the research team, a professor of ethics at a Swiss university who was part of a separate Swiss Ethical Board for ethical advice. The confidentiality of the participants was ensured and prior to the interviews, written informed consent was obtained from every participant."

x26-ii) Outline informed consent procedures

"The confidentiality of the participants was ensured and prior to the experiment, written informed consent was obtained from every participant."

X26-iii) Safety and security procedures

No safety and security procedures were necessary, and: "The confidentiality of the participants was ensured and prior to the experiment, written informed consent was obtained from every participant. "

X27-i) State the relation of the study team towards the system being evaluated

No conflicts declared
